# Lateral Breast and Axillary Reconstruction With Pedicled Parascapular Flap

**Published:** 2018-09-26

**Authors:** Christina Kapsalis, Jared Davis, Bradon J. Wilhelmi

**Affiliations:** ^a^School of Medicine, University of Louisville, Louisville, Ky; ^b^Division of Plastic and Reconstructive Surgery, Department of Surgery, University of Louisville, Louisville, Ky

**Keywords:** parascapular flap, lateral breast and axillary reconstruction, adjuvant radiotherapy, breast cancer, immediate breast reconstruction

## Abstract

**Objective:** Immediate flap reconstruction following mastectomy in patients who may require adjuvant radiation therapy can be controversial. However, exposure of vital structure or defects too large for primary closure may necessitate immediate utilization of flaps. In this setting, both functional and cosmetic outcomes must be considered. **Methods:** We describe a case in which the patient required a wide excision of the axillary skin and a partial mastectomy, with a resulting large axillary and lateral breast skin defect. The ipsilateral pedicled parascapular flap was used for immediate reconstruction, with primary donor site closure. **Results:** Seven years postoperatively, she remained disease free, contracture free, with near-normal shoulder range of motion, and good cosmesis. **Conclusions:** Our case supports prior studies that have demonstrated the parascapular flap to have low donor site morbidity compared with other harvest sites. Our patient did not suffer from loss of functional range of motion or limitation in physical activity. It also demonstrates the flap's utility for lateral breast and axillary coverage and its durability in the setting of adjuvant radiation therapy.

The parascapular flap is versatile and has multiple advantages. These include constant vascular anatomy, territory suitable for large wound coverage, potential primary donor site closure, and practicality as either a pedicled flap or a free flap.^1^ It has been described for coverage of upper and lower extremity defects^2^^-^4 and scalp defects.^5^ Mayou et al^6^ also determined that an islandized parascapular flap can be appropriate for defects of the shoulder, axilla, breast, chest wall, and anterior neck.

The scapular and parascapular flaps have gained popularity from their constant vasculature, pedicle length, desirable thickness, ease of primary donor site closure, potential for combination with other flaps, and lack of musculoskeletal deficits.^2^^,^^7^^,^^8^ The parascapular flap has further advantages when compared with the scapular flap. These advantages include its large potential donor skin surface area and favorability for harvest with the patient in lateral position.^7^ Although there can sometimes be difficulty achieving primary closure of the donor site, the axial orientation of the flap usually allows for primary donor site closure in flaps up to 15-cm wide.^2^^,^^7^

Clinical applications of the scapular flap are broad and have been well described.^9^ The cutaneous scapular artery has been found to have a relatively constant anatomy and serves as the main pedicle of the free scapular flap. dos Santos defined the free scapular flap as “a cutaneous area of a fusiform shape that is transverse to the longitudinal axis of the body.”^9^^(p601)^ Nassif et al^2^ soon followed these findings by describing the anatomy and clinical applications of a parascapular flap. The parascapular flap, like the free scapular flap, is based on the circumflex scapular artery. However, the pedicle of the parascapular flap is the vertically oriented cutaneous parascapular artery.^2^ The vessels of the subscapular system are rarely adversely affected by atherosclerosis, which could play a role in the reliability of these flaps.^10^

A study exploring parascapular donor site morbidity completed in 2007 by Roll et al^11^ found no deformity of the shoulder girdle. In this same study, only 2 patients suffered from range-of-motion limitations. The study ultimately concluded that donor site morbidity of parascapular flaps is tolerable and is lower when compared with other harvest sites.^11^ In 2013, Klinkenberg et al^12^ compared anterolateral thigh, lateral arm, and parascapular free flaps. They observed that the parascapular flap had the lowest donor site morbidity and the highest patient satisfaction.^12^ Our case supports these findings, as our patient did not suffer from any range-of-motion deficits or limitations in physical activity. It also demonstrates the flap's utility for axillary coverage in the setting of a lateral breast and axillary skin and soft tissue defect, and its durability and retained shoulder range of motion after adjuvant radiation therapy.

## METHODS

A 57-year-old woman presented with a 2-year history of subcutaneous nodules in her right anterior axilla. On examination, there was no ectopic breast tissue, dominant mass, nipple retraction, or discharge. The right axilla had multiple small, firm subcutaneous nodules that occupied most of the hair-bearing skin. Mammography demonstrated suspicious micro-calcifications in the right axilla and faint calcifications in the superior right breast, and magnetic resonance imaging of the breast did not reveal any abnormality. Stereotactic breast biopsy was negative for tumor; however, biopsy of the skin and subcutaneous nodules demonstrated high-grade ductal carcinoma in situ (DCIS) with central necrosis.

**Figure 1:**
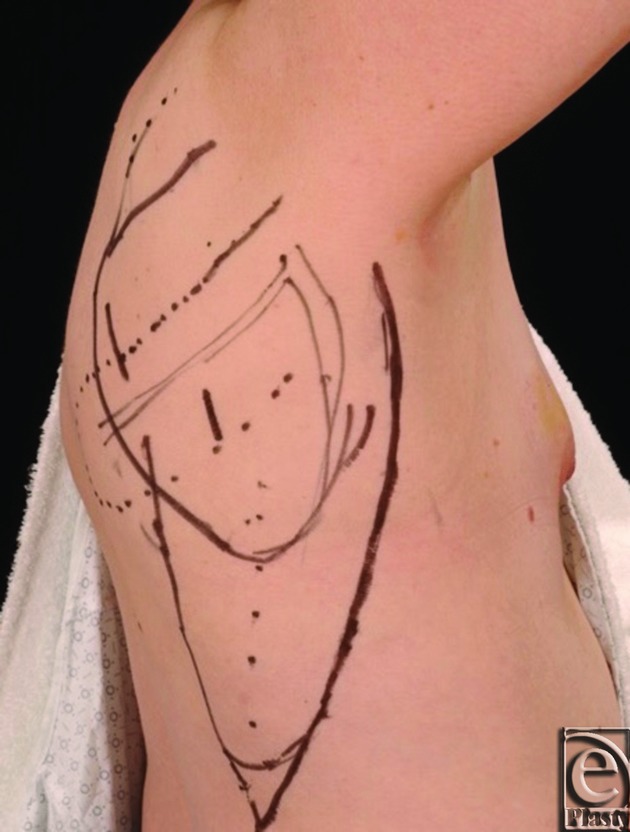
Preoperative parascapular flap design.

**Figure 2:**
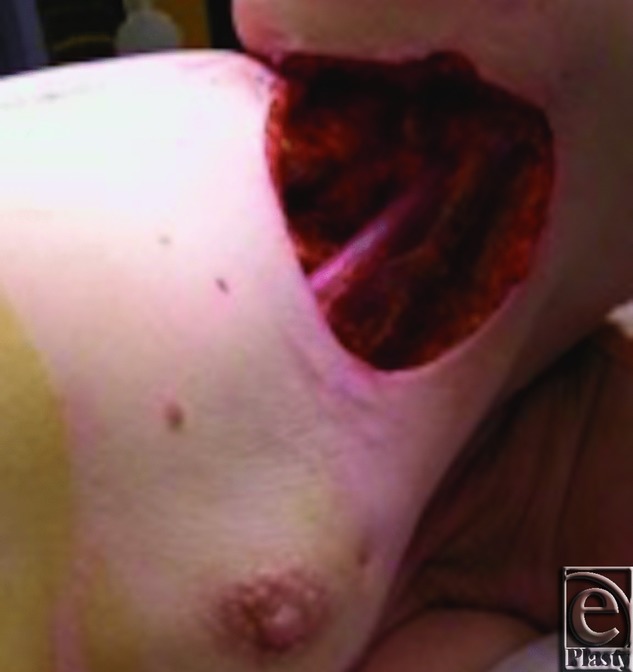
Defect of axilla and lateral breast.

**Figure 3:**
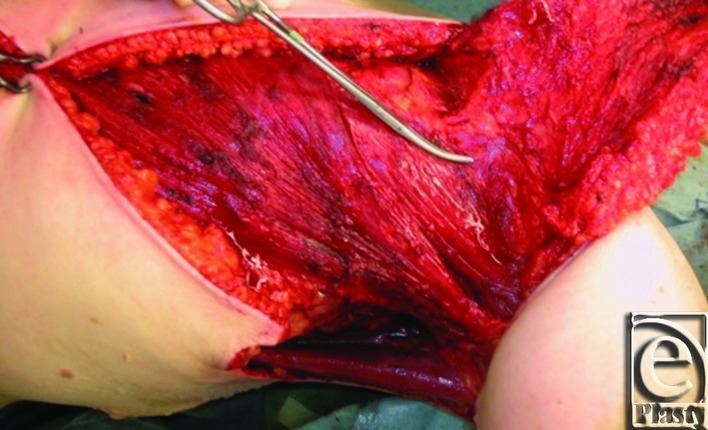
Elevated parascapular flap.

Prior to resection by the oncologic team, the planned parascapular flap was marked (Fig 1). She underwent a wide excision of the axillary skin en bloc with partial mastectomy including the tail of Spence and sentinel lymph node biopsy. After the resection (Fig 2), the ipsilateral pedicled parascapular flap was used for reconstruction (Fig 3) and the donor site was closed primarily.

## RESULTS

**Figure 4:**
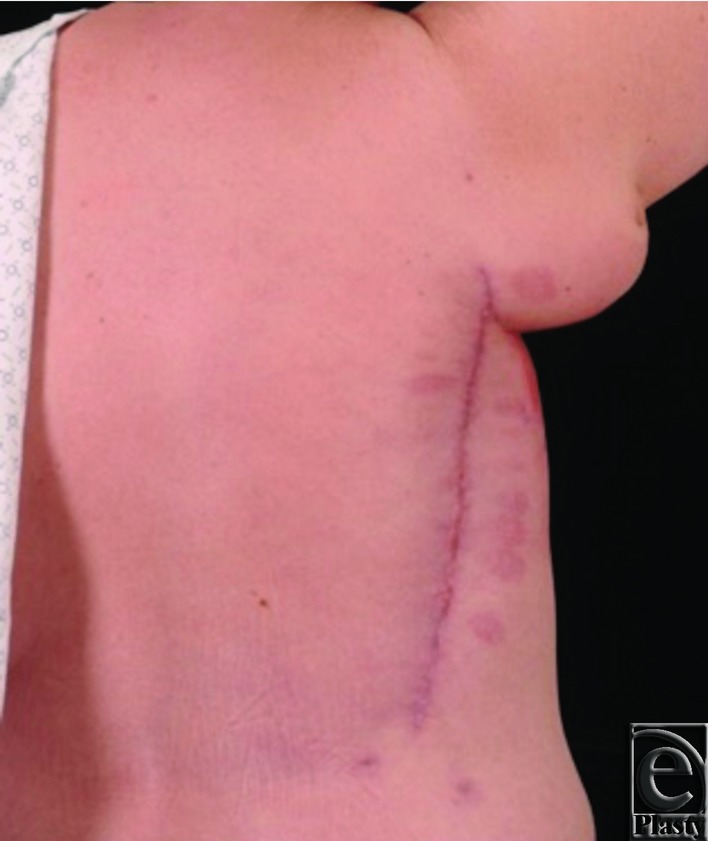
Seven-year postoperative follow-up.

Her immediate postoperative course was uneventful. Final pathology showed DCIS in the right axillary skin with a 2-mm focus of invasive ductal carcinoma in the partial mastectomy specimen, widely negative margins, and negative sentinel nodes. Hormone status was ER+ and PR− with Her2Neu overexpression. The patient received adjuvant treatment with tamoxifen, followed by anastrozole, and whole breast and axillary radiotherapy, which resulted in increased flap edema. However, there were no wound complications and she maintained active range of motion of her shoulder. Seven years postoperatively, she remained disease free, contracture free, with normal shoulder range of motion, and good cosmesis (Fig 4).

## DISCUSSION

The need for adjuvant radiotherapy complicates decision making when performing immediate flap reconstruction. Postreconstruction radiotherapy is associated with increased potential for complications including inferior aesthetic outcome, asymmetry, contracture, and hyperpigmentation.^13^^,^^14^ Despite such controversy, the efficacy of free and pedicled flaps for immediate reconstruction in the setting of likely adjuvant radiation therapy has previously been demonstrated.^15^ In our case, immediate flap coverage was needed to cover the axillary contents, and this outweighed flap risks posed by radiation. Wound contractures over joints can be problematic, and the axilla is especially prone to contractures secondary to contour and postoperative postitioning.^16^ Fibrosis and contracture of this anatomic site carry the morbidity of decreased shoulder range of motion and loss of function.

At 7 years postoperatively, the patient still maintained full functional range of motion and no contractures were present (Fig 4). The flap remained bulky with moderate contour abnormality, but the patient was satisfied with the result and elected to forego any debulking or revision. It is noteworthy to mention that the flap thickness might have contributed to its durability in the setting of postoperative radiotherapy.

This case demonstrates the benefits and versatility of this flap. The relatively thin, hairless skin paddle and primary donor site closure make it cosmetically favorable. It also provides stable soft tissue coverage without the need for muscle sacrifice, allows a quick functional recovery, and withstands adjuvant radiotherapy without significant contracture.
